# Minimally invasive non-surgical therapy (MINST) in stage III periodontitis patients: 6-month results of a split-mouth, randomised controlled clinical trial

**DOI:** 10.1007/s00784-023-04994-4

**Published:** 2023-04-04

**Authors:** Alja Cmok Kučič, Rok Gašperšič

**Affiliations:** 1 Public Health Centre Celje, Gregorčičeva 5, 3000 Celje, Slovenia; 2 Department of Oral Medicine and Periodontology, Faculty of Medicine, University of Ljubljana, Hrvatski Trg 6, 1000 Ljubljana, Slovenia; 3 Department of Oral Medicine and Periodontology, Dental Clinic, University Clinical Centre, Hrvatski Trg 6, 1000 LjubljanaLjubljana, Slovenia

**Keywords:** Periodontitis, Minimally invasive, Scaling and root planning, Nonsurgical periodontal debridement

## Abstract

**Objectives:**

To determine if minimally invasive non-surgical therapy (MINST) outperforms classical non-surgical periodontal therapy for stage III periodontitis with primarily suprabony (horizontal) type defects.

**Materials and methods:**

In a split-mouth randomised controlled trial, 20 patients’ dental quadrants were randomly assigned to MINST or classical non-surgical treatment. The primary outcome variable was the number of sites with probing pocket depth ≥ 5 mm and BOP. Treatment method, tooth type, smoking status, and gender were evaluated using a multivariate multilevel logistic regression model.

**Results:**

After 6 months, the percentage of sites with PD ≥ 5 mm and BOP that healed (MINST = 75.5%; control group = 74.1%; *p* = 0.98), and the median number of persisting sites (MINST: 6.5, control group: 7.0; *p* = 0.925) were similar in both groups. In the test and control groups, respectively, median probing pocket depths (2.0 mm vs. 2.1 mm) and clinical attachment level (1.7 mm vs. 2.0 mm) changed significantly (*p* < 0.05) but similarly. Significantly less gingival recession occurred in the MINST group’s deep molar pockets compared to the control group (*p* = 0.037). Men (OR = 0.52, *p* = 0.014) and non-molars (OR = 3.84, *p* 0.001) had altered odds for healing of sites with PD ≥ 5 mm and BOP.

**Conclusions:**

MINST reduces gingival recession associated with molar teeth, although it performs similarly to traditional non-surgical therapy in treating stage III periodontitis with predominately horizontal-type defects.

**Clinical relevance:**

MINST performs similarly to non-surgical periodontal therapy in stage III periodontitis with predominantly suprabony defects.

**Trial registration:**

Clinicaltrials.gov (NCT04036513) on June 29, 2019.

**Supplementary Information:**

The online version contains supplementary material available at 10.1007/s00784-023-04994-4.

## Introduction

The initial periodontal treatment aims to minimise supra/subgingival plaque, calculus deposits, and bacterial load through behavioural adjustments and risk factor management, supported by non-surgical debridement techniques. As the second stage therapeutic measure, surgical intervention or extractions are recommended for more advanced periodontitis [1]. Angular bony (intrabony) defects, for example, are considered to be associated with an increased risk of disease progression and tooth loss, necessitating treatment beyond non-surgical intervention [2, 3]. Regenerative techniques have gradually supplanted the traditional resective surgical eradication of such defects, resulting in a clinical and radiographic improvement of periodontal attachment [4]. Minimally invasive techniques that emphasise conservative flap design, primary wound closure, and blood cloth preservation are now the standard therapeutic approach [5–7]. Compared to conventional therapy, minimally invasive techniques have reduced morbidity, a shorter postoperative time, less recession, and greater patient-reported acceptability [8]. However, due to the common inclusion of bone grafts, barrier materials, and growth factors [9], even minimally invasive surgeries carry a risk of morbidity, poor predictability, and higher costs [10].

Nibali et al. devised a minimally invasive non-surgical periodontal treatment protocol (MINST). According to their retrospective case series evaluation, using MINST as a non-surgical periodontal treatment for intrabony defects resulted in decreased probing depth (PD), increased clinical attachment level (CAL), and radiographically determined bone fill [3, 9, 11]. The use of ultrasonic devices with delicate tips, small curettes with longer terminal shanks and thinner blades, and magnification has been recommended as a part of the MINST protocol to enable thorough subgingival debridement with minimal tissue trauma [3]. As a recent study comparing minimally invasive surgical and non-surgical procedures demonstrated no clinical difference in the healing of intrabony defects [5, 9], MINST may be regarded as an effective alternative to surgical treatment [12]. It is postulated that a similar healing process following MINST and minimally invasive surgical procedures results from superior blood clot stability that is, through healing processes, replaced by a new connective tissue attachment [3].

However, when treating advanced periodontitis, supra-alveolar (horizontal) defects are found to be three to nine times more prevalent than intrabony defects. Their ability for regeneration is limited, and therapeutic effectiveness remains a challenge [13]. In addition, mechanical disruption of subgingival biofilm in deep periodontal pockets accompanied by horizontal or vertical bone loss is complex and frequently results in residual pockets [1].

The study by Iorio-Siciliano et al. [14] is the only randomised controlled trial (RCT) addressing the treatment of deep pockets with MINST alone or in conjunction with NaOCl gel, irrespective of the type of the defect. Since the study by Chung et al. [15] was a pilot study, to our knowledge, no published randomised controlled trials utilising the principles of MINST as an alternative to standard non-surgical debridement for the treatment of advanced periodontitis with primarily supraalveolar bone loss are available. Therefore, the aim of this study was to determine if, for the treatment of stage III periodontitis with predominant supraalveolar defects associated with horizontal bone loss, MINST would result in a lower number of sites with PD ≥ 5 mm and BOP that are commonly perceived as requiring further treatment in clinical practice [16]. Our hypothesis was that by using MINST, the number sites with PD ≥ 5 mm and BOP remaining after non-surgical periodontal therapy would be reduced compared to conventional non-surgical periodontal therapy.

## Materials and methods

A 6-month, single-centre, split-mouth, randomised controlled clinical trial was conducted. The National Medical Ethical Committee of the Republic of Slovenia granted ethical approval (No. 0120–595/2018/4). The clinical protocol was also submitted to Clinicaltrials.gov (No. NCT04036513). Before taking part, all subjects were given a description of the proposed treatment and gave their informed and written consent. The study was carried out following the Helsinki Declaration’s principles.

### Study population

All included patients were referred by their general dentists to the Department of Oral Medicine and Periodontology, University Dental Clinic of Ljubljana, Slovenia, for periodontal assessment and treatment. Between March 2019 and November 2021, 20 male or female participants were explicitly selected as participants in this study of 207 consecutively evaluated individuals.

Age 18 to 70 years, stage III periodontitis (grade B/C according to the AAP/EFP classification 2018), presence of 20 teeth for stable occlusion (excluding third molars), and equally distributed periodontal pockets on both sides of the jaw and between molar and non-molar teeth, with at least 9 non-molars in the upper arch, were the inclusion criteria. Exclusion criteria included the following: periodontal treatment in the last 12 months, the presence of prosthetic restorations, implants, endo-perio lesions, pregnancy, lactation, and systemic medical conditions that could affect the progression of periodontal disease or healing (i.e. HIV/AIDS, cancer, diabetes mellitus, diseases of bone metabolism, radiation therapy, chemotherapy, immunosuppressive therapy, antiepileptics, calcium antagonists, and nonsteroidal anti-inflammatory drugs). Less than 10 cigarettes smoked per day was not considered an exclusion criterion.

### Clinical and radiographic examination

At baseline and follow-ups, an experienced, masked, calibrated examiner (R. G.) performed a comprehensive periodontal examination of all patients using a manual Williams probe (POW6, Hu-Friedy, Chicago, Illinois, USA). At 6 sites of each tooth, the following periodontal parameters were measured: absence/presence of plaque on tooth surfaces using a dichotomous plaque index, absence/presence of bleeding upon gentle probing around the gingival crevice, probing pocket depth (PD), gingival recession (REC), and absence/presence of BOP provoked by gentle applying of a probe to the bottom of a sulcus/pocket. The percentage of plaque-positive sites was expressed as FMPS [17], and the percentage of gingival bleeding sites as FMBS [18]. The intraclass correlation coefficients for PD and REC were greater than 0.90 and the kappa values for FMPS, FMBS, and BOP were greater than 0.95, indicating excellent reproducibility in a calibration exercise involving 10 patients with periodontitis stage III/IV and measurements repeated after 1 week. PD and REC were added to determine clinical attachment loss (CAL). Tooth mobility was measured and graded [19]. Furcation involvement [20] was evaluated for each tooth; however, furcation involvement was omitted from further analysis. Since further CAL is commonly anticipated and surgical therapy is thought necessary for sites measuring ≥ 5 mm with persistent BOP, the number of such sites per patient was determined. Full-mouth periapical radiographs were acquired using the long cone paralleling technique to determine the type of alveolar bone defect.

### Clinical intervention

After a baseline examination, each of the 20 patients received two 90-min sessions of cause-related periodontal non-surgical treatment from the same therapist (A. C. K.) within 7 days, beginning with motivation and instruction on proper oral hygiene. Both dental arches’ left and right sides were randomly assigned to one of two treatment modalities. Randomisation was accomplished by using a computer program formula to generate the random allocation sequence number (Microsoft Office Excel, Microsoft Corporation, Redmond, W, USA). Then, a random allocation number was assigned for each side of the dentition, with an even or odd number corresponding to the test or control group. The treatment allocation cards were sealed in envelopes until the clinical procedure. To maintain blinding and allocation concealment, randomisation was carried out, and the envelopes containing specific treatment modalities were handled by a third party who was not involved in the patient’s treatment or clinical assessment. As a result, the patient and the evaluator were blinded throughout the treatment. The treatment protocol in the test quadrants adhered to the MINST concepts described by Nibali et al. [3]. Under local anaesthesia (Ultracain, Hoechst, France) and 3.5 × magnification loupes (ExamVision, Sams, Denmark), supra- and subgingival deposits were thoroughly debrided. Specific thin and delicate tips (P20, P21L, P21R, PN3, NSK, Tochigi, Japan) in the power-driven piezo-electric device (Varios970Lux, NSK, Tochigi, Japan) were used to minimise trauma to soft tissues, supplemented by manual Gracey mini-curettes (“micro-mini five”, Hu-Friedy, Chicago, IL, USA). There was no subgingival rinsing after the procedure to stimulate the formation of a stable blood clot. The control quadrants had conventional supra- and subgingival deposits removed under local anaesthesia (Ultracain, Hoechst, France) without any additional magnification devices. Again, the majority of the time, a power-driven piezo-electric ultrasonic scaling device (Varios970Lux, NSK, Tochigi, Japan) with scaling tips (G5, G8, NSK, Tochigi, Japan) was used to debride subgingival biofilm and calculus, supplemented by manual standard Gracey curettes (Hu-Friedy, Chicago, IL, USA). Finally, both sides of the dental arch were polished with a mechanical brush and professional toothpaste (Proxyt RDA 7, Ivoclar Vivadent, Lichtenstein). Chair-side time was recorded from when local anaesthesia was administered until the end of the debridement procedure.

The subjects were recalled 1, 3, and 6 months after treatment. Oral hygiene instructions were reinforced at each appointment, and at 3- and 6-month follow-ups, a full-mouth periodontal examination was performed using the same protocol as at baseline and the same type of periodontal probe (POW6, Hu-Friedy, Chicago, Illinois, USA). In addition, all sites with PD ≥ 5 mm and BOP sites were re-instrumented with the same treatment modality. At the 6-month follow-up, the buccal surfaces of the teeth were stimulated using a dental syringe and compressed air for 1 s to induce pain. The discomfort was localised to each quadrant and recorded as present or absent [21].

### Statistical analysis

Since further clinical attachment loss is expected at sites with PD ≥ 5 mm and persistent BOP after non-surgical periodontal treatment, surgical intervention is usually indicated [16]. Sites demonstrating both features (PD ≥ 5 mm + BOP) were counted and their number was defined as the primary outcome measure. A sample size of 16 participants would be sufficient to confirm a statistically significant difference of 2 sites with PD ≥ 5 mm + BOP with a standard deviation of 2, 90% statistical power, and statistical significance set at *p* < 0.05. The sample size was increased by 15% to account for non-parametric statistical tests. Secondary outcome measures were changes in the median number of PD, REC, CAL, and BOP, the number of sites with PD ≥ 5 mm + BOP within molars and non-molars, and the proportion of sites with PD ≥ 5 mm + BOP that healed. Mann–Whitney *U* test was used to evaluate the intergroup differences of numerical clinical parameters. The prevalence differences within and between groups were analysed using Fisher’s exact test. Post-treatment numerical clinical parameters were compared with the baseline using Wilcoxon’s signed rank test for intragroup analyses. A multivariate multilevel logistic regression model evaluated the effects of gender, smoking, treatment modality, and tooth type on the number of persisting sites with PD ≥ 5 mm + BOP set as the dependent variable, considering sites as nested with teeth and teeth nested within patients. The random intercept was individually predicted for every subject and tooth, allowing for variability in the probability of site healing with regard to each tooth and subject. A *p* value < 0.05 was set to accept a statistically significant difference. SPSS v. 26 was used for the analyses.

## Results

**Fig. 1:**
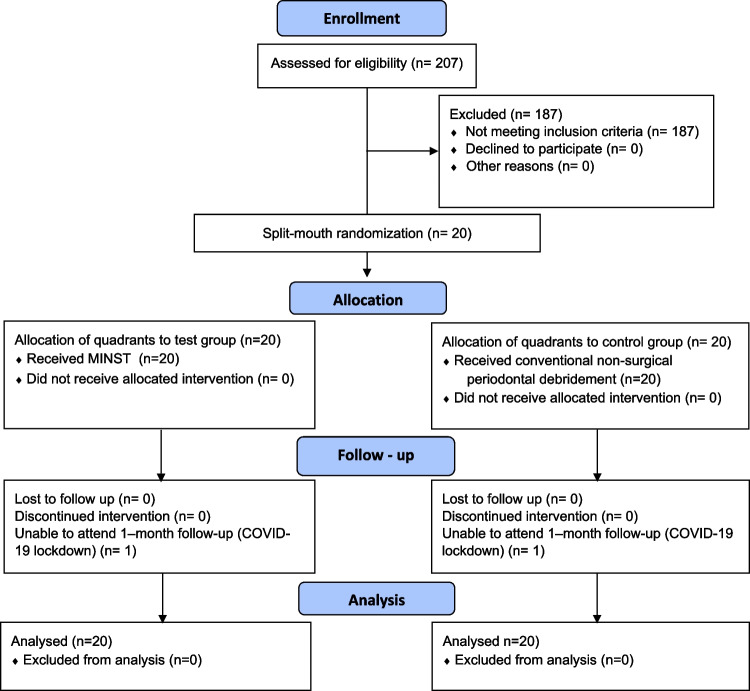
CONSORT flow chart

In total, none of the 20 subjects who received baseline treatment withdrew. Due to COVID-19 pandemic lockdown, one patient could not be screened after 1 month. Figure 1 depicts the patients’ enrolment, allocation to treatment, disposition, and analysis status. Table 1 shows the population’s baseline characteristics. The average age of the subjects was 43.15 ± 8.80, 7 were men (35%), and 13 were women (65%). The percentage of smokers was 20% (4 participants).

**Table 1:** Baseline characteristics of the population

Mean age (years: mean (SD))	43.15 (8.80)
Males (*n* (%))	7 (35)
Women (*n* (%))	13 (65)
Smokers (*n* (%))	4 (20)

*SD*, standard deviation

### Clinical outcomes

**Fig. 2:**
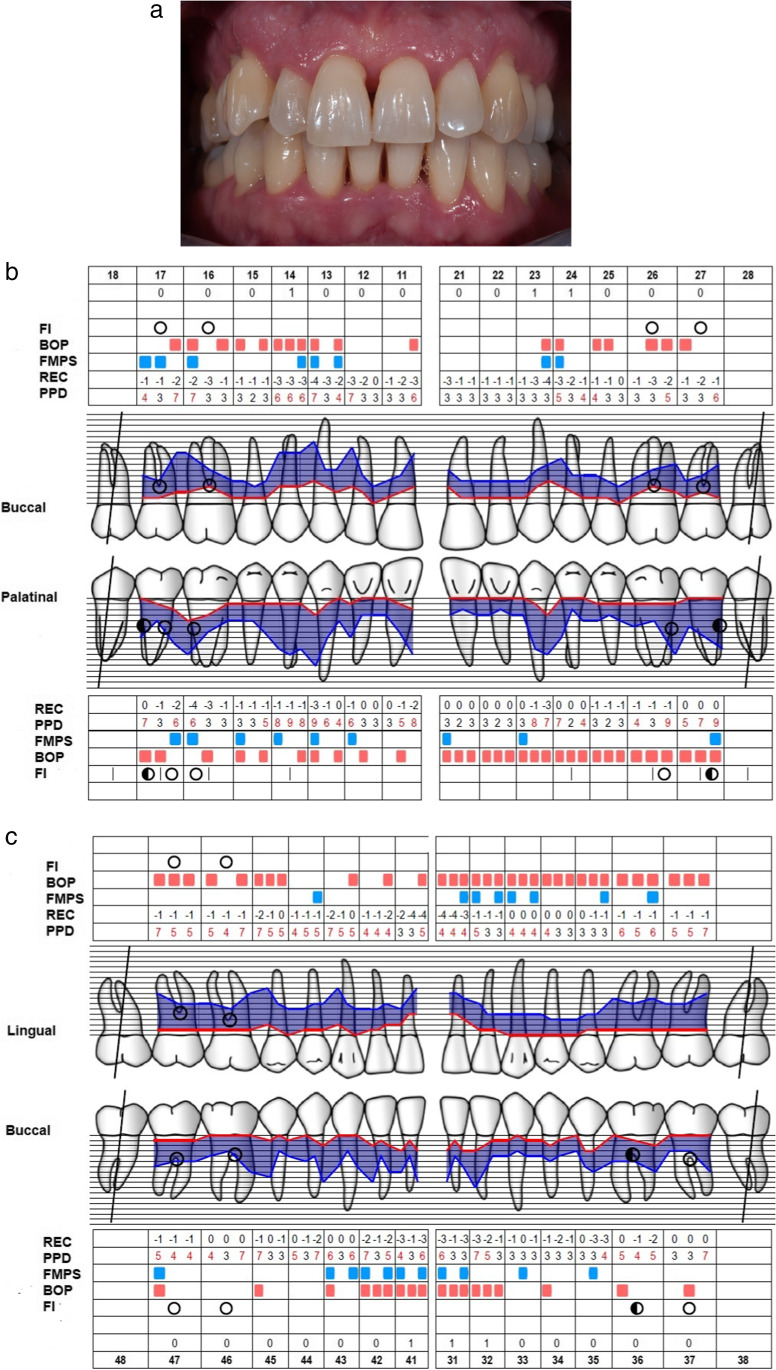
**a** Clinical baseline characteristics of the representative patient. **b** Periodontal parameters of the maxillary dental arch. **c** Periodontal parameters of the mandibular dental arch

**Fig. 3:**
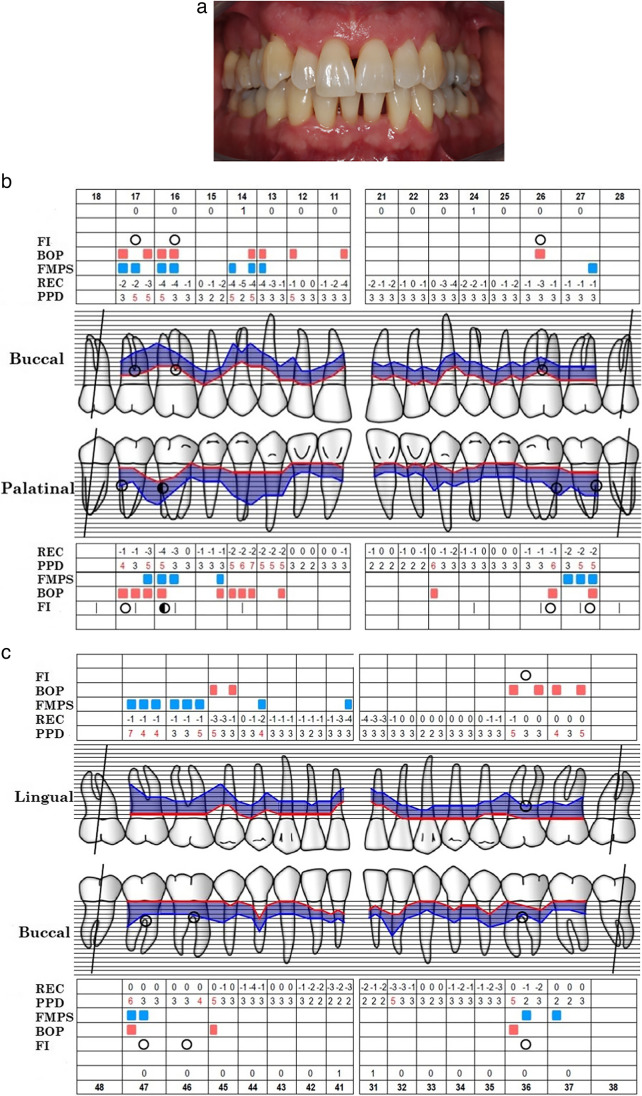
**a** Clinical characteristics of the representative patient after 6 months of follow-up. The right quadrants were treated with MINST and the left quadrants were treated with the standard non-surgical approach. **b** Periodontal parameters of the maxillary dental arch. **c** Periodontal parameters of the mandibular dental arch

Table 2 displays the median values (Me) and interquartile ranges (IQR) of the primary outcome measures in relation to tooth type, treatment modality, and timeline. On initial examination, the median number of sites with PD ≥ 5 mm + BOP in the MINST group was 26.5 (IQR: 12.0–31.0) and 27.0 (IQR: 17.5–21.5) in the control group. The test group had Me 16.5 (IQR: 11.00–22.0) sites with PD ≥ 5 mm + BOP on non-molars and Me 11.0 (IQR: 5.5–12.0) sites with PD ≥ 5 mm + BOP on molars, whereas the control group had Me 15.5 (IQR: 10.5–21.0) sites with PD ≥ 5 mm + BOP on non-molars and Me 9.0 (IQR: 6.5–11.0) sites with PD ≥ 5 mm + BOP on molars. Three months after treatment, there was a statistically significant intragroup reduction in median number of sites with PD ≥ 5 mm + BOP comparing all teeth (Me: 7.0 (IQR: 3.5–10.0) in the test, Me = 6.0 (IQR: 3.5–9.0) in the control group), non-molars (Me = 2.0 (IQR: 1.0–4.0) in the test, Me = 2.5 (IQR: 1.0–4.0) in the control group), and molars (Me: 4.0 (IQR: 2.0–6.5) in the test, Me: 3.5 (IQR: 1.5–6.0) in the control group). At the 6-month follow-up, the number of sites with PD ≥ 5 mm + BOP remained almost unchanged (Figs. 2 and 3). No statistically significant intergroup differences were found between the two groups in all parameters.

**Table 2:** The number of sites with PD ≥ 5 mm and BOP in relation to tooth type, treatment modality and timeline Me (IQR)

	All teeth			Non-molars			Molars		
	MINST	Control group	*p*	MINST	Control group	*p*	MINST	Control group	*p*
T0	26.5 (21.0; 31.0)	27.0 (17.5; 21.5)	0.968	16.5 (11.0; 22.0)	15.5 (10.5; 21.0)	0.904	11.0 (5.5; 12.0)	9.0 (6.5; 11.0)	0.512
T3	7.0* (3.5; 10.0)	6.0* (3.5; 9.0)	0.82	2.0* (1.0; 4.0)	2,5* (1.0; 4.0)	0.841	4.0* (2.0; 6.5)	3.5* (1.5; 6.0)	0.814
T6	6.5* (3.0; 10.5)	7.0* (2.5; 10)	0.925	2.0* (1.0; 5.0)	1.0* (0.5; 4.5)	0.512	4.0* (1.5; 5.0)	3.5* (1.0; 6.5)	0.947

*T0*, baseline; *T3*, 3-month follow-up; *T6*, 6-month follow-up

^*^Statistically significant change in comparison to baseline (T0) *p* < 0.001

Table 3 shows the baseline median and interquartile range (IQR) of PD, CAL, and REC at sites with PD ≥ 5 mm + BOP relative to tooth type, pocket depth, and treatment modality and their changes at 3- and 6-month follow-up. Clinical parameters in both groups were comparable at baseline and their changes were comparable at 3- and 6-month follow-up, but there were statistically significant intragroup differences within both groups (before and after). At baseline, the median PDs in the test and control groups were 6.04 mm (IQR: 5.62–6.27) and 5.98 mm (IQR: 5.53–6.30), respectively. These values were reduced by 1.84 mm (IQR: 1.47–2.30) and 2.09 mm (IQR: 1.31–2.34) 3 months after treatment. Non-molar teeth and deep pockets showed the greatest improvement. Further changes in PD were negligible at 6 months compared to 3-month follow-up, with no statistically significant differences between treatment types. Except for REC, all periodontal parameters improved 3 and 6 months after treatment in both groups. From 3- to 6-month follow-up, recession associated with deep pockets of molar teeth in the MINST group (Me: 0.00 mm (IQR: − 0.30–0.38)) was statistically significantly lower than in the control group (Me: 0.32 mm (IQR: 0.00–1.00)) (*p* = 0.037). Table 4 depicts FMPS and FMBS at baseline and both follow-ups. All intergroup differences in all parameters were minor and statistically insignificant. There were no statistically significant differences in any clinical outcomes at the 6-month follow-up compared to the 3-month follow-up.

**Table 3:** Baseline clinical outcomes and changes at 3-month (T3) and 6-month follow-up (T6) in relation with tooth type, pocket depth and treatment modality Me (IQR)

	T3														
	All sites			Shallow pockets (5–6 mm)						Deep pockets (≥ 7 mm)					
				Non-molars			Molars			Non-molars			Molars		
	MINST	Control	*p*	MINST	Control	*p*	MINST	Control	*p*	MINST	Control	*p*	MINST	Control	*p*
PD (mm) baseline	6.04 (5,62; 6,27)	5.98 (5.53; 6.30)	0.862	5.20 (5.11; 5.36)	5.25 (5.17; 5.48)	0.172	5.08 (5.00; 5.20)	5.22 (5.00; 5.50)	0.454	7.66 (7.13; 8.32)	7.27 (7.00; 8.32)	0.057	7.88 (7.44; 8.11)	8.00 (7.00; 8.21)	0.937
∆ PD (mm) T3	1.84* (1.47; 2.30)	2.09* (1.31; 2.34)	0,695	1.71* (1.55; 2.00)	1.94* (1.25; 2.34)	0.483	1.44* (0.62; 2.00)	1.33* (0.75; 1.75)	0.918	3.13* (2.12; 3.71)	2.81* (1.89; 3.76)	0.783	2.25* (1.19; 2.91)	2.34* (1.97; 3.00)	0.611
∆ PD (mm) T6	0.16 (− 0.10; 0.43)	0.14 (− 0.27; 0.52)	0.892	0.22 (0.08; 0.30)	0.25 (− 0.53; 0.42)	0.977	0.13 (− 0.75; 0.6)	0.00 (− 0.6; 0.43)	0.977	0.21 (− 0.06; 0.66)	0.45 (− 0.26; 1.05)	0.580	0.38 (− 0.4; 1.00)	0.00 (− 0.33; 1.0)	0.657
CAL (mm) baseline	7.00 (6.44; 7.40)	6.73 (6.47; 7.64)	0.82	6.00 (5.71; 6.50)	6.00 (5.38; 6.50)	0.759	6.00 (5.50; 6.88)	6.30 (5.58; 7.33)	0.538	8.67 (8.07; 9.78)	8.20 (7.81: 9.39)	0.565	8.59 (8.19; 9.36)	8.75 (7.91; 9.75)	0.937
∆ CAL (mm) T3	1.63* (1.05; 2.07)	1.92* (1.35; 2.24)	0.297	1.55* (0.94; 1.55)	1.77* (1.0; 2.18)	0.895	1.20* (0.6; 2.0)	1.50* (1.0; 2.0)	0.559	2.88** (1.97; 3.42)	2.73* (1.65; 3.45)	0.890	1.58* (1.19; 2.0)	1.91* (1.0; 2.83)	0.409
∆ CAL (mm) T6	0.07 (− 0.23; 0.25)	0.07 (− 0.24; 0.40)	0.735	0.00 (− 0.25; 0.42)	+ 0.07 (− 0.63; 0.41)	0.919	0.00 (− 0.5; 0.5)	+ 0.44 (− 0.40; 0.89)	0.298	0.20 (− 0.23; 0.51)	0.45 (− 0.41; 0.78)	0.346	0.31 (− 0.30; 1.08)	+ 0.48 (− 0.50; 1.27)	0.071
REC (mm) baseline	0.94 (0.34; 1.30)	0.93 (0.38; 1.24)	0.678	0.78 (0.50; 1.40)	0.75 (0.25; 1.00)	0.405	1.00 (0.20; 1.75)	1.00 (0.17; 1.83)	0.692	0.84 (0.15; 1.89)	1.00 (0.25; 1.00)	0.775	0.84 (0.17; 1.10)	1.00 (0.00; 1.35)	0.847
∆ REC (mm) T3	** + **0.20** (0.01; 0.36)	** + **0.16** (0.00; 0.30)	0.698	0.00 (− 0.33; 0.50)	+ 0.08 (− 0.12; 0.47)	0.334	+ 0.08 (− 0.16; 0.25)	0.00 (− 0.75; 0.25)	0.492	+ 0.25** (0.00; 0.60	0.00 (− 0.20; 0.50)	0.195	+ 0.38** (0.00; 0.82)	+ 0.40** (0.00; 1.00)	0.861
∆ REC (mm) T6	+ 0.08 (− 0.05; 0.25)	+ 0.13 (− 0.10; 0.29)	0.799	0.00 (− 0.12; 0.43)	0.00 (− 0.19; 0.25)	0.456	0.00 (− 0.17; 0.50)	+ 0.16** (0.00; 1.00)	0.166	0.00 (− 0.25; 0.34)	0.00 (− 0.20; 0.00)	0.736	0.00* (− 0.30; 0.38)	+ 0.32** (0.00; 1.00)	0.037

*PD* probing depth, *CAL* clinical attachment level, *REC* recession, *∆ PD* difference in PD relative to previous evaluation, *∆ CAL* difference in CAL in relative previous evaluation, *∆ REC* difference in REC relative to previous evaluation, − improvement, + deterioration

^*^Statistically significant change < 0.001 and ** < 0.05 in comparison to baseline

**Table 4:** FMPS, FMBS in relation to time (T3: 3-month follow-up, T6: 6-month follow-up) and treatment modality Me (IQR) (%)

	Baseline			T3			T6		
	MINST	Control	*p*	MINST	Control	*p*	MINST	Control	*p*
FMPS	47.5 (28.3; 62.8)	41.0 (30.3; 59.5)	0.925	21.0 (9.0; 29.0)	29.0 (9.3; 32.8)	0.461	20.5 (15.5; 28.5)	24.0 (9.3; 32.8)	0.779
∆ FMPS				28.5** (1.3; 46.3)	18.0*(5.3; 30.3)	0.565	4.0 (− 12.5; 10.0)	+ 1.0 (-3.8; 19.3)	0.284
FMBS	12.0 (0.0; 23.8)	9.0 (0.0; 27.0)	0.880	0.0 (0.0; 5.8)	0.0 (0.0; 13.8)	0.369	1.5 (0.0; 18.8)	0.0 (0.0; 8.0)	0.620
∆ FMBS				6.5** (0.0; 22.0)	3.5** (0.0; 26.3)	0.820	0.0 (0.0; 14.5)	0.0 (− 1.5; 7.5)	0.112

*FMPS* full-mouth plaque score, *FMBS* full-mouth bleeding score; + deterioration

^*^Statistically significant change < 0.001 and ** < 0.05 in comparison to T3

A total of 54 mobile teeth were identified, with 24 in the control group and 30 in the experimental group. Four teeth in the test group and four in the control group demonstrated mobility grade 2, while the remaining teeth demonstrated mobility grade 1. After 6 months, the mobility of 29 teeth was reduced, including 10 teeth in the control group and 19 teeth in the test group. All eight teeth with a mobility grade of 2 were downgraded to grade 1, and of the 46 teeth with a mobility grade of 1, 21 became non-mobile. After 6 months of recovery, the mobility of 10/24 (42%) teeth in the control group and 19/30 (63%) teeth in the test group was reduced (*p* = 0.19).

Table 5 shows the proportion of healed sites, i.e. sites with baseline PD ≥ 5 mm and BOP that changed after treatment (PD < 5 mm and no BOP), relative to non-molar/molar tooth. After 6 months, 75.5% of all sites with PD ≥ 5 mm and BOP in the MINST group and 74.1% in the control group were healed. Non-molars had a higher proportion of such sites than molars. Even though there were minor intergroup differences, they were not statistically significant.

**Table 5:** Proportion of sites with baseline PD ≥ 5 mm and BOP that have healed after 6 months

	MINST (*n* = 20)	Control (*n* = 20)
Baseline		
Proportion of DS at baseline (Me (IQR)) (%)	32.0 (25.0; 37.0)	32.0 (21.0; 39.0)
6-month follow-up		
Proportion of HS (Me (IQR)) (%)	75.5 (66.1; 85.7)	74.1 (69.2; 88.2)
Proportion of HS—molars (Me (IQR)) (%)	63.6 (58.3; 72.7)	61.1 (40.9; 84.6)
Proportion of HS—non-molars (Me (IQR)) (%)	87.9 (77.3; 90.9)	93.5 (78.6; 95.2)

*DS* diseased site (PD ≥ 5 mm + BOP), *HS* healed site (PD < 5 mm and no BOP)

When the other variables were controlled for, the multivariate multilevel logistic regression model revealed that healing of sites with PD ≥ 5 mm + BOP was negatively associated with molar tooth type and gender (Table 5). Probing sites on non-molars had a statistically significant (*p* 0.001) higher odds ratio (OR) for healing than probing sites on molars (OR: 3.84–95% CI: 2.36–6.37) and a lower OR (*p* = 0.014) in men (OR: 0.52–95% CI: 0.31–0.87) (Table 6).

**Table 6:** Association between treatment modality, tooth type, smoking, gender and site healing (multivariate multilevel logistic regression model)

	OR (95% CI)	*p*
Men	0.52 (0.31; 0.87)	0.014*
Smoking	1.02 (0.64; 1.64)	0.923
Treatment modality	1.07 (0.66; 1.76)	0.78
Non-molar	3.84 (2.36; 6.27)	< 0.001*

*OR* odds ratio, *CI* confidence interval

^*^Statistically significant change

### Patient-reported outcomes

Conventional therapy and the MINST procedure required comparable average chair-side times (62 ± 11 and 64 ± 14 min for conventional and MINST, respectively). After treatment, none of the patients complained of pain or required pain medication. In addition, 17/40 quadrants of the test group and 19/40 quadrants of the control group demonstrated teeth with post-treatment sensitivity to provocation on compressed air 3 months after treatment (*p* = 0.82). After 6 months, these figures decreased to 6/40 and 8/40, respectively (*p* = 0.78). All four patients who smoked gave up the habit during their 6-month recovery. Aside from sensitivity, no adverse effects were reported.

## Discussion

The primary goal of mechanical debridement as a part of non-surgical periodontal therapy is biofilm removal. According to the findings, gaining access to instruments becomes more difficult as PD progresses, as manual curettes cannot reach the bottom of the periodontal pocket in 75% of patients with advanced periodontitis [22]. Similarly, only 43% of surfaces are cleaned after scaling for periodontal pockets between 4 and 6 mm deep, whereas this figure drops to 32% for PD > 6 mm [23]. For this reason, hand instruments with a thinner profile and/or longer shanks and ultrasonic tips with dimensions matching the periodontal probe to allow easier access into deeper pockets, furcations, and grooves were developed [24] to be used during MINST described by Nibali et al. [12]. The primary goal of our split-mouth, randomised, controlled clinical study was to evaluate if MINST, when compared to conventional non-surgical debridement, reduces the number of sites with residual PD ≥ 5 mm and BOP, which typically indicates the need for further intervention, in patients with stage III periodontitis with the predominantly supra-alveolar bone loss after 6 months. Because of the reduced tissue trauma caused by microsurgical instruments and magnifying agents, we expected a lower proportion of residual pockets in less accessible regions and better healing in supraalveolar compartments. A multilevel logistic regression analysis revealed no link between the treatment mode and the recovery of DS. This was consistent with the absence of statistically significant differences between groups in primary and secondary outcomes (probing pocket depth reduction and clinical attachment gain). As no further improvement in healing capacity was attained by MINST, we can assume that the development of a more stable blood clot may be hampered by the structural constraints of horizontal bone loss (avascular root surface), permitting only healing by establishing a long junctional epithelium [25–29]. This is congruent with the findings of Nibali et al., who observed an increase of 0.5 mm in suprabony defect depth due to bone remodelling following MINST treatment of intrabony defects [3, 12]. The literature also does not provide a single universally accepted MINST protocol. A new modified MINST protocol [9] was proposed in 2019 that proposes subpapillary access, local anaesthesia without adrenaline, the abolition of mini-curettes, and an initial re-evaluation not before 6 months of recovery. We can speculate that the vasoconstrictive effect of a regular anaesthetic solution containing adrenaline may have reduced blood clot stability and that the mini-curettes may damage most coronal parts of the papillae. Future research should assess the MINST technique using anaesthetics that do not contain adrenaline and debridement done by power-driven ultrasonic devices.

Nevertheless, after 3 months of therapy, the periodontal condition significantly improved in both groups and remained stable after 6 months. The proportion of healed sites 6–8 months following conventional non-surgical debridement was 74% in the most recent systematic review by Suvan et al. [30] and 74–77% in the study by Wennström et al. [31]. These values are consistent with those seen in our test group (75.5%) and the control group (74.1%). Similarly, using MINST for deep pockets, Iorio-Siciliano et al. [14] found a 74.3% reduction in the number of diseased sites after 6 months. In their study, however, only patients with mild to moderate disease progression rates (grades A and B) were included, and those with multi-rooted teeth or furcation involvement were excluded. In yet another clinical trial, participants who underwent active periodontal treatment using the MINST protocol had a mean proportion of pocket closure of 71.6% after just 2 months [32].

The median PD decreased from 6.04 to 4.11 mm in the test group and from 5.98 to 4.14 mm in the control group after 3 months without further improvement after 6 months. The difference between the test and control groups was not statistically significant, although it was smaller in the test group (1.84 mm) than in the control group (2.09 mm). Wennström et al. [31] also found a reduction in pocket depth that was identical to that ascertained by MINST (1.8 mm), while other studies reported even better results [14, 32]. The most recent systematic review [30] found that regardless of tooth type, the expected PD reduction after 3/4 months was 1.5 mm in shallow and 2.6 mm in deep pockets, which follows our observation of more significant PD reduction in deep pockets, than in shallow pockets.

A gingival recession is one of the most common complaints from patients after non-surgical interventions, especially in high aesthetic areas [5, 33]. After 3 months, both groups had a slight but statistically significant recession compared to the initial condition (0.20 mm in the test and 0.16 mm in the control group). The literature suggests a recession increase could be as much as 1 mm for shallow pockets and 2 mm for deeper ones [34]. A noteworthy, albeit maybe coincidental, finding of the study is that gingival recession in the region of the molars was reduced when the MINST technique was employed as opposed to the conventional method. This may be due to the minimally invasive, gentle treatment of fragile soft tissues to prevent damage during instrumentation and maintain blood flow [35, 36].

As previously stated, the clinical outcomes did not change following the second treatment cycle. These findings are consistent with research by Badersten et al. [37] that demonstrated no difference between a single episode of non-surgical treatment and repeated instrumentation at 3-month intervals in terms of clinical indicators. Approximately 50% of pockets with an initial PD of 7 mm remained diseased, predominantly molars with root furcation involvement, deep pockets, and intrabony defects [31]. Following mechanical re-instrumentation, only 11 to 16% of all sites that respond poorly to the initial treatment achieve a satisfactory treatment outcome [33].

False positive interpretations and cause-and-effect conclusions are more likely to occur in statistical analyses that fail to account for the fact that diseased sites are not isolated entities. Therefore, the results of the multilevel multivariate logistic regression that considers nesting, which showed a lower odds ratio for healing residual sites on molars and, surprisingly, in men, are also noteworthy findings of this study. It is well-known that molars have a lower success rate than other teeth due to anatomic features, demanding plaque control, and limited access to professional care [38]. On the other hand, no conclusive evidence supports the hypothesis that men and women respond to periodontal treatment differently. Our intervention included more women (65%) than men, which may reflect a gender disparity in dental care seeking and professional help. Even though there were no differences between males and females in our study, it is well known that males frequently have higher probing depth (PD) values and clinical attachment loss [39, 40]. Contrary to our expectations, smoking did not hinder sites with PD ≥ 5 mm and BOP recovery. Due to the presence of four smokers (20% of the sample), the efficacy of the MINST technique may have been compromised, as vasoconstriction and insufficient blood flow caused by smoking may limit the periodontal tissue’s regenerative capacity. Therefore, it is logical to assume that the MINST approach would have a greater impact on nonsmokers. Nonetheless, as the subanalysis conducted after the exclusion of smokers (data not shown) failed to identify any significant differences between the groups, we believe that a small number of smokers did not considerably influence the results of our investigation. In addition, it should be underlined that comprehensive and continuous motivation regarding the importance of quitting smoking was sufficient to persuade all four patients to quit during the 6-month recovery period.

We sought to evaluate the periodontal tissue response to two distinct clinical procedures within the same dentition (split-mouth design) because the immune response and healing capacity differ between individuals. Therefore, only patients with periodontal pockets on both the molar and non-molar sites of both jaws were considered for the study. The high percentage of patients who were not included (187 out of 207 examined) demonstrates how challenging it was to meet this requirement. Furthermore, our sampling strategy resulted in a higher number of diseased sites and more advanced forms of periodontitis at baseline compared to other studies evaluating the influence of non-surgical periodontal treatment on pocket closure [41, 42]. The disease’s progression rate and the generalised pattern were reflected in the lower age of the included patients than in comparable studies that used MINST protocol [14, 32].

**Fig. 4:**
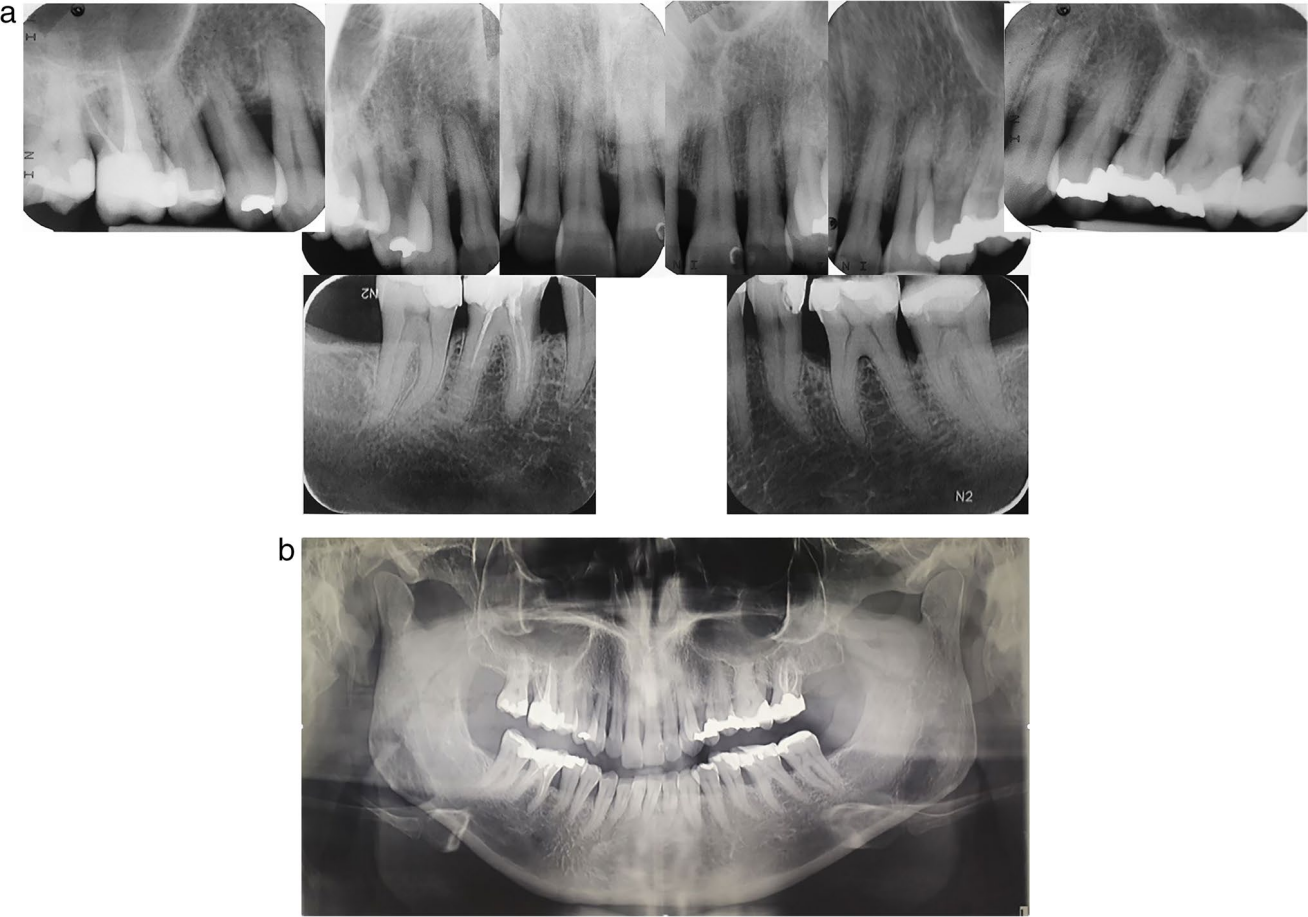
**a** Local radiographs of a representative patient. **b** Orthopantomogram of a representative individual

Since it is known that periodontitis can affect distinct jaw regions differently, a split-mouth design may limit the investigation’s scope. It seems sensible to test the MINST protocol using two parallel subject groups in the future. Due to the possibility of poorer response to the MINST protocol in smokers, it would be sensible to evaluate the effects of MINST on the treatment of suprabony periodontal defects in non-smokers and smokers separately, in addition, to evaluate a more recent version of MINST employing an anaesthetic without adrenaline and the use of curettes. To increase external validity, it would be necessary to study the efficacy of MINST in a broader range of patient populations, including patients with various healing disorders and non-academic clinical settings. Furthermore, the patient follow-up period may be extended to 1 year, and treatment outcomes may also be examined radiographically (Fig. 4).

## Conclusion

MINST and standard non-surgical periodontal debridement resulted in significant but comparable decreases in the number of sites with PD ≥ 5 mm + BOP and other clinical parameters. Furthermore, in a multilevel, multivariate logistic regression model, male gender and molar teeth were related to a higher number of residual sites with PD ≥ 5 mm + BOP. Therefore, within the scope of this study, it is reasonable to state that the MINST regimen is a clinically beneficial initial non-surgical treatment for stage III periodontitis characterised by predominantly supraalveolar bone loss, yielding results comparable to the classical treatment approach.

## Supplementary Information

Below is the link to the electronic supplementary material.

Supplementary file1 (DOC 219 KB)

## Data Availability

The data that support the findings are avaliable on reasonable request from corresponding author.
